# Cerebral Hemodynamic and White Matter Changes of Type 2 Diabetes Revealed by Multi-TI Arterial Spin Labeling and Double Inversion Recovery Sequence

**DOI:** 10.3389/fneur.2017.00717

**Published:** 2017-12-22

**Authors:** Yelong Shen, Bin Zhao, Lirong Yan, Kay Jann, Guangbin Wang, Junli Wang, Bao Wang, Josef Pfeuffer, Tianyi Qian, Danny J. J. Wang

**Affiliations:** ^1^School of Medicine, Shandong Medical Imaging Research Institute, Shandong University, Jinan, China; ^2^Laboratory of FMRI Technology (LOFT), Keck School of Medicine, Mark and Mary Stevens Neuroimaging and Informatics Institute, University of Southern California (USC), Los Angeles, CA, United States; ^3^Siemens Healthcare, Erlangen, Germany; ^4^Siemens Healthcare, MR Collaborations NE Asia, Beijing, China

**Keywords:** arterial spin labeling, cerebral blood flow, bolus arrival time, type 2 diabetes, white matter hyperintensity, double inversion recovery

## Abstract

Diabetes has been reported to affect the microvasculature and lead to cerebral small vessel disease (SVD). Past studies using arterial spin labeling (ASL) at single post-labeling delay reported reduced cerebral blood flow (CBF) in patients with type 2 diabetes. The purpose of this study was to characterize cerebral hemodynamic changes of type 2 diabetes using a multi-inversion-time 3D GRASE pulsed ASL (PASL) sequence to simultaneously measure CBF and bolus arrival time (BAT). Thirty-six patients with type 2 diabetes (43–71 years, 17 male) and 36 gender- and age-matched control subjects underwent MRI scans at 3 T. Mean CBF/BAT values were computed for gray and white matter (GM and WM) of each subject, while a voxel-wise analysis was performed for comparison of regional CBF and BAT between the two groups. In addition, white matter hyperintensities (WMHs) were detected by a double inversion recovery (DIR) sequence with relatively high sensitivity and spatial resolution. Mean CBF of the WM, but not GM, of the diabetes group was significantly lower than that of the control group (*p* < 0.0001). Regional CBF decreases were detected in the left middle occipital gyrus (*p* = 0.0075), but failed to reach significance after correction of partial volume effects. BAT increases were observed in the right calcarine fissure (*p* < 0.0001), left middle occipital gyrus (*p* < 0.0001), and right middle occipital gyrus (*p* = 0.0011). Within the group of diabetic patients, BAT in the right middle occipital gyrus was positively correlated with the disease duration (*r* = 0.501, *p* = 0.002), BAT in the left middle occipital gyrus was negatively correlated with the binocular visual acuity (*r* = −0.408, *p* = 0.014). Diabetic patients also had more WMHs than the control group (*p* = 0.0039). Significant differences in CBF, BAT, and more WMHs were observed in patients with diabetes, which may be related to impaired vision and risk of SVD of type 2 diabetes.

## Introduction

As the most common metabolic disorder in humans, type 2 diabetes is characterized by insulin resistance and relative insulin deficiency. Patients with type 2 diabetes are prone to developing long-term complications affecting the eyes, kidneys, heart, blood vessels, and nerves. These complications can affect the life quality and increase the mortality of patients with type 2 diabetes (1). Diabetes retinopathy is a common complication of diabetes with a prevalence of 24.5% in those with known type 2 diabetes (2, 3). Even with the availability of effective treatment, it remains one of the leading causes of visual loss (4–6).

Type 2 diabetes can affect the central nervous system (7), resulting in cognitive decline, metabolic, and vascular changes (8) affecting both the micro and macro vasculature (9). It has been hypothesized that hypoperfusion may underlie impaired cognitive function associated with type 2 diabetes. Reduced cerebral blood flow (CBF) has been reported using single-photon emission computed tomography (SPECT) (10) and arterial spin labeling (ASL) (11, 12). In brain MRI, patients with type 2 diabetes exhibit more cortical and subcortical atrophy (13) and more deep white matter (WM) lesions (14, 15) than control subjects. In addition, impaired blood–brain barrier (BBB) function has been implicated in cerebral effects of type 2 diabetes (16).

Arterial spin labeling is a non-invasive technique, which labels the protons contained in the arterial blood water by exciting them through radiofrequency pulses, without the need of exogenously administered contrast media (17, 18). This is especially beneficial for patients with type 2 diabetes, who are frequently ineligible for undergoing infusion of gadolinium-based contrast agents, due to the chronic diabetic complications affecting kidneys (chronic renal failure). However, to date, there are only a limited number of ASL studies having studied CBF changes in patients with diabetes. A recent longitudinal study using pseudo-continuous ASL found decreased global CBF as well as regional CBF in the resting-state default mode, visual, and cerebellum networks in patients with type 2 diabetes. Greater decrease in longitudinal CBF values at these regions over a 2-year span was associated with worse cognitive functions, and higher baseline insulin resistance (12).

As another hemodynamic parameter, bolus arrival time (BAT) also provides clinically relevant information regarding the status of cerebrovasculature but has not been explored in type 2 diabetes. In this study, we applied a multi-inversion-time (mTI) 3D GRASE pulsed ASL (PASL) sequence to simultaneously measure CBF and BAT in a cohort of subjects with type 2 diabetes, and compared the results with those of matched control subjects. This mTI 3D GRASE PASL protocol can not only detect both CBF and BAT defects in patients with diabetes but also make more accurate quantification of CBF by including BAT measurement. Based on existing literature, we hypothesize that there are decreased CBF and/or prolonged BAT affecting the whole brain (e.g., the WM) and specific cortical regions (e.g., the visual cortex) that are associated with clinical characterization and/or behavioral performance in subjects with type 2 diabetes.

White matter lesions such as white matter hyperintensities (WMHs) are commonly observed in cerebral small vessel disease (SVD) secondary to diabetes (19, 20). In the present study, a 3D double inversion recovery sequence (3D-DIR) was applied for the detection of WMHs by simultaneously suppressing the signal of cerebrospinal fluid and WM (21). This double inversion recovery (DIR) sequence has been shown to be able to accurately delineate the location and the number of WM lesions in multiple sclerosis (22), and hippocampal pathology (23). The high sensitivity and small slice thickness of 3D-DIR make it suitable for imaging WMHs in type 2 diabetes. We hypothesize that there are greater WMHs in subjects with type 2 diabetes compared to controls that are associated with clinical characterization and/or behavioral performance.

## Materials and Methods

### Subjects

The study was approved by the institutional ethical committee. Informed consent was obtained from all volunteers and patients after the nature of the study had been fully explained to them. Forty-one patients with type 2 diabetes and 41 gender- and age-matched control subjects participated in this study. Between September 2014 and November 2016, patients with type 2 diabetes were recruited in the outpatient department of Shandong Provincial Hospital and control participants were recruited from the spouses or acquaintances of the patients.

For inclusion, type 2 diabetic patients (diagnosed by oral glucose tolerance test) had to be 40–80 years of age, have a disease duration of at least 1 year, and be functionally independent. Exclusion criteria for all participants included a psychiatric or neurological disorder that could influence cognitive functioning, history of alcohol or substance abuse, or history of dementia. All participants did not have a history of stroke and no large confluent WMHs (>20 mm) was found in the DIR image as this lesion may affect the perfusion result. Only small WMHs (≤20 mm) were allowed, which are defined as small lesions located in the WM with the characteristics of hyperintensity on FLAIR, without cavitation (24). Lesions in the subcortical gray matter (GM) or brainstem were not included as WMHs. Participants whose MRI quality was poor due to patients’ movement were excluded from further analyses (*n* = 5), resulting in a total of 72 participants (36 patients with type 2 diabetes: age 43–71 years old, 17 male; and 36 controls: age 42–76, 14 male). For control participants, exclusion criteria additionally included fasting blood glucose ≥7.0 mmol/L.

The demographic and clinical information about the presence of vascular risk factors was assessed by means of a standardized interview and by physical and laboratory examinations. Blood pressure was measured at nine fixed time points during the day with an automatic blood pressure device. Hypertension was defined as a mean systolic blood pressure above 160 mmHg, a mean diastolic pressure above 95 mmHg, or the use of antihypertensive medication.

### MR Imaging

All data were collected on an MAGNETOM Skyra 3 T MR scanner (Siemens, Erlangen, Germany) using a 20-channel head-neck coil. The MRI exam consisted of multi-TI ASL, T1 MPRAGE, and 3D fast spin-echo double inversion recovery sequence (SPACE-DIR). Multi-TI ASL images were acquired with a background-suppressed 3D GRASE PASL sequence with the following parameters: TR/TE = 4,600/22 ms, FOV = 220 mm × 220 mm, slice thickness = 4 mm, voxel size = 3.4 mm × 3.4 mm × 4.0 mm, 36 slices, bolus length = 700 ms, 16 TIs from 480 to 4,080 ms with a step of 225 ms, and total acquisition time = 5:09 min including an M0 scan. The CBF, BAT, and residual error maps were calculated in-line on the scanner using vendor software. MPRAGE was acquired in a sagittal orientation with 1 mm isotropic resolution (FOV = 230 mm × 230 mm, 192 slices, TR/TE = 1,900 ms/2.58 ms, TI = 900 ms, flip angle = 9°) in 4:59 min. The imaging parameters for SPACE-DIR were: TR/TE = 7,500/319 ms, 144 slices, slice thickness = 1.4 mm, FOV = 230 mm × 230 mm, matrix size = 192 × 192, IR-Delays 450/3,000 ms, total acquisition time = 6:17 min. All participants wore earplugs, and their heads were immobilized with a vacuum bean bag pillow, padded earmuffs, and a plastic bar across the bridge of the nose.

### Neurobehavioral and Binocular Visual Acuity Test

The Montreal Cognitive Assessment (MOCA) was performed on all participants, which has been shown to be a better screening tool for mild cognitive impairment (MCI) in type 2 diabetes population. Compared to Standardized Mini-Mental Status Exam, MOCA has a better sensitivity in diagnosing MCI (25). Binocular visual acuity was measured by standard logarithmic visual acuity chart through bare eye sight.

### Data Processing

The model of Buxton et al. (26) was fitted with a nonlinear fitting algorithm (vendor software) to obtain quantitative CBF and BAT maps. The model function used the following parameters: λ = 0.9, T1 of arterial blood = 1,650 ms, T1 of brain tissue = 1,330 ms, and bolus length = 700 ms. CBF and BAT maps were co-registered to the structural MRI, and then normalized to the Montreal Neurological Institute (MNI) space and spatially smoothed with a 6 mm FWHM kernel, using Statistical Parametric Mapping 8.^1^

First of all, the structural MRI was segmented into GM and WM. The masks of the GM and WM were determined by the threshold of greater than 0.6 for GM and 0.8 for WM on tissue segmentation results using SPM8. The whole brain mask was determined by combining those of the GM and WM. Mean CBF and BAT values were extracted from the GM, WM and whole brain masks, respectively, and compared between the diabetes group and the control group using two-sample *T*-test.

A voxel-based analysis was then applied to compare regional CBF and BAT differences between diabetes and control groups, using a two-sample two-sided *t*-test. The threshold was set to the false discovery rate corrected *p* < 0.05 with a cluster size of 50 voxels to localize those brain regions with significant differences between the two groups.

Corrections of potential partial volume effects (PVE) on regional CBF differences were performed according to the methods described in Du et al. (27). The GM and WM probability maps obtained through voxel-based morphometry (28) of SPM8 were down-sampled to the spatial resolution of perfusion MRI. We assumed that perfusion of WM is 40% of that of GM based on previous PET study (29). To account for PVE, ASL-CBF were corrected according to the following equation: Icorr = Iuncorr/(GM + 0.4 × WM), where Icorr and Iuncorr are the corrected and uncorrected intensities, and GM and WM are the tissue probabilities, respectively.

White matter hyperintensities were detected and quantified on 3D DIR images according to consensus criteria by Wardlaw et al. (30). Participants with large confluent WMHs were excluded (>20 mm) because they may affect CBF. Only the number of WMHs on the SPACE-DIR images was assessed by two neuro-radiologists independently who were blinded to the origin of data and the mean value was used for analysis. Each neuro-radiologist counted the number of WMHs 3 times from axial, coronal, and sagittal views of 3D DIR images, respectively, and the mean number was reported. We defined five levels according to the number of the WMHs: Level 1 (0), Level 2 (1–3), Level 3 (4–10), Level 4 (11–20), Level 5 (≥21). The inter-rater agreement was calculated for grading of WMHs using Cohen’s kappa in SPSS Statistics 17.0 (IBM, Chicago, IL, USA).

### Statistical Analysis

Differences in neuropsychological performance and MR variables between patient and control groups were analyzed with multivariable linear regression adjusted for age and sex. Statistical maps were overlaid onto a high-resolution brain template in the standard MNI space using XJVIEW software.^2^ To investigate the relationship between the abnormal cerebral perfusion patterns (i.e., BAT and CBF) and clinical characteristics, the regional values of BAT and CBF in the identified brain areas were correlated with the illness duration using Pearson’s correlation. The statistical analyses were performed using GraphPad Prism 5 software (GraphPad Software, Inc.).

To determine whether CBF, BAT, and the number of WMHs in diabetic patients was related to the disease duration and binocular visual acuity, we correlated mean CBF and BAT in the clusters displaying significant group differences with the disease duration and binocular visual acuity across diabetic subjects. Pearson correlation was also performed between CBF in the significant clusters and MOCA scores. The Kolmogorov–Smirnov and Shapiro–Wilk tests were used to test the normal distribution of variables, and Spearman correlation was applied if the data did not follow normal distribution.

## Results

### Participants’ Characteristics

Table 1 shows the demographic and clinical information of diabetes and control groups, respectively. The two groups were well balanced in terms of age, sex, and level of education. The diabetic subjects were significantly heavier than controls (*p* = 0.035), with a higher BMI (*p* = 0.091). There were no significant differences in terms of comorbid conditions (e.g., hypertension or hyperlipidemia) between the two groups.

**Table 1:** Patient characteristics.

Participant characteristics	Type 2 diabetes patients	Control subjects	P value
Sex (male/female)	17/19	14/22	0.482
Age (years)	57.61 ± 6.21	56.19 ± 6.84	0.361
Race	Asian	Asian	
Education (years)	9.08 ± 1.52	9.81 ± 2.94	0.2
Height(m)	1.67 ± 0.08	1.652 ± 0.10	0.444
Weight(kg)	72.38 ± 9.48	66.58 ± 13.05	0.035*
BMI (kg/m^2^)	25.99 ± 2.88	24.44 ± 4.61	0.091
Diabetes duration (years)	5.43 ± 4.88		
Recent fasting glucose (mmol/l)	8.00 ± 1.04	5.7 ± 0.33	<0.001***
Use of insulin (%)	18 (50)		
Hypertension (yes/no)	20/16	13/23	0.159
Hyperlipidemia (yes/no)	9/27	8/28	0.785
White matter hyperintensities (%)	29 (81)	14 (39)	0.004**
Binocular visual acuity	4.61 ± 0.25		
Montreal Cognitive Assessment	25.69 ± 0.86	26.03 ± 0.84	0.101

Data shown are means and SD or proportions (%); *p < 0.05; **p < 0.005; ***p < 0.001.

### CBF and BAT Changes of the Diabetes Group

**Figure 1:**
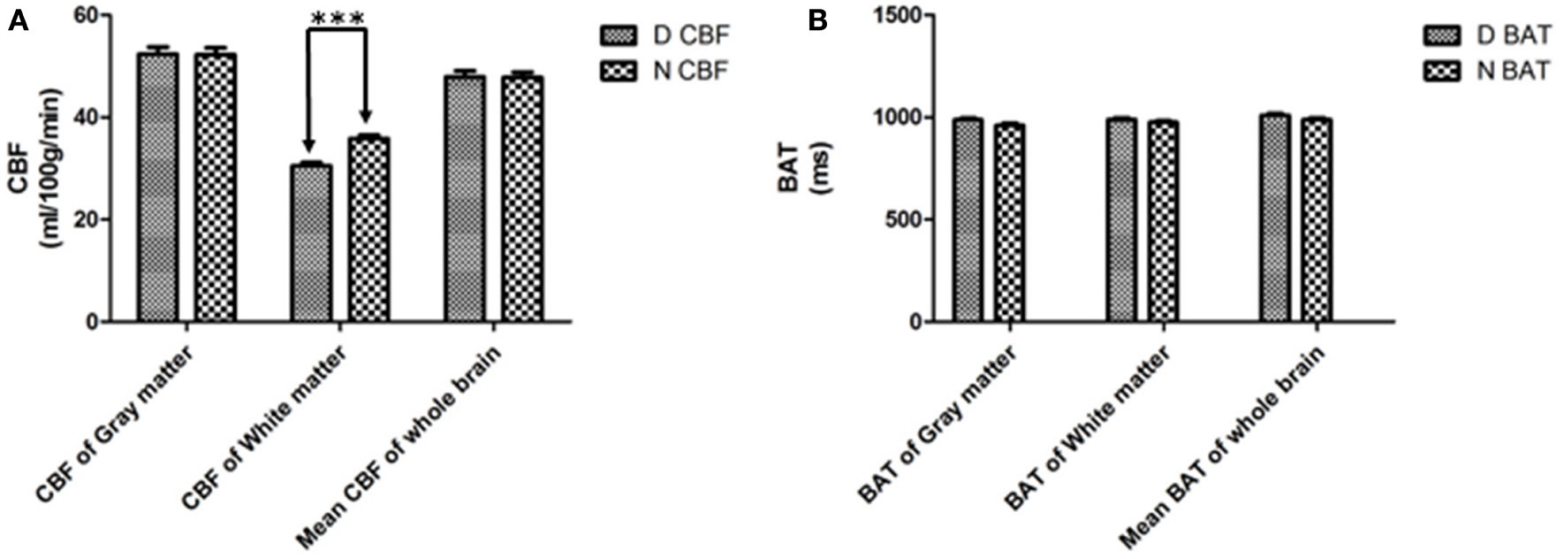
Cerebral blood flow (CBF) **(A)** and bolus arrival time (BAT) **(B)** of white matter, gray matter and the mean value of the whole brain. D = patients with type 2 diabetes, N = the normal control. ***p < 0.001.

The mean global CBF of the diabetes and control group was 47.90 ± 7.05 and 47.29 ± 6.40 ml/100 g/min, respectively. The mean global BAT of the diabetes and control group was 1,009.0 ± 52.3 and 988.1 ± 54.4 ms, respectively. No significant differences were found between the two groups (Figure 1B) except that the CBF of the WM of the diabetes group (30.48 ± 3.99 ml/100 g/min) was significantly lower than that of the control group (35.82 ± 3.86 ml/100 g/min) (*p* < 0.0001) (Figure 1A).

**Figure 2:**
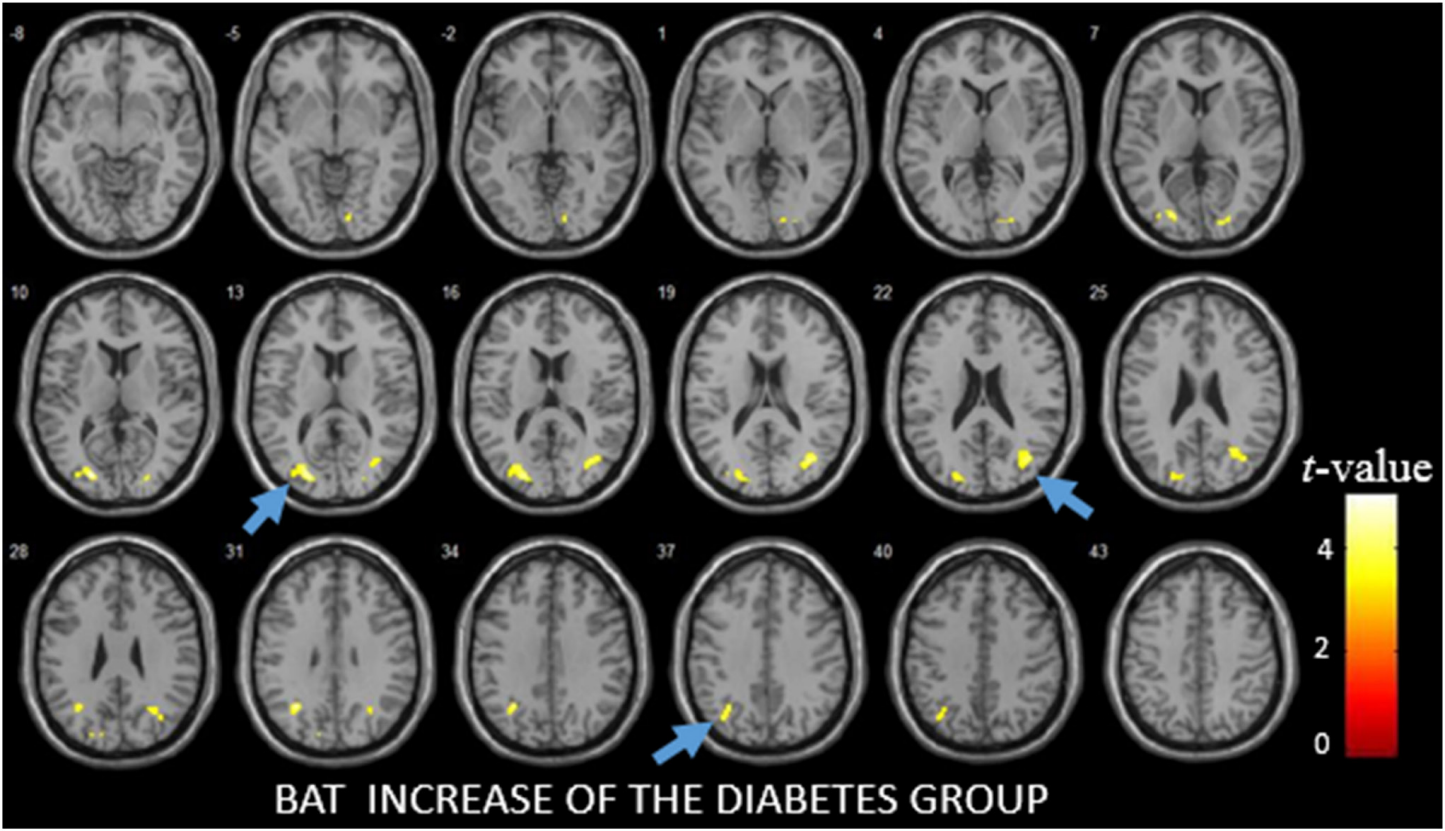
The difference of bolus arrival time (BAT) between patients with diabetes and the control group as detected by voxel-based analysis. The light blue arrows show three areas with the most significant differences in the BAT maps.

Before the PVE correction, significant CBF decreases were detected in the left middle occipital gyrus (*p* = 0.0075). After the PVE correction, no significant CBF changes were detected between the diabetes and control group. BAT increases were observed in the right calcarine fissure and surrounding cortex (*p* < 0.0001), two clusters in left middle occipital gyrus (*p* < 0.0001) and right middle occipital gyrus (*p* = 0.0011) (Figure 2; Table 2).

**Table 2:** Areas with the most significant differences in the bolus arrival time (BAT) maps as obtained from the voxel-based analysis.

	BAT increase of the diabetes group		
	ROI	No. of voxels	*p*-Value
Brain areas	Right calcarine fissure and surrounding cortex	66	<0.0001
	(14 −86 −2)		
	Left middle occipital gyrus	252/97	<0.0001/<0.0001
	(−22 −84 12/−30 −60 32)		
	Right middle occipital gyrus	222	0.0011
	(38 −70 20)		

*The coordinates refer to the standard Montreal Neurological Institute space*.

### WMHs of the Two Groups

**Figure 3:**
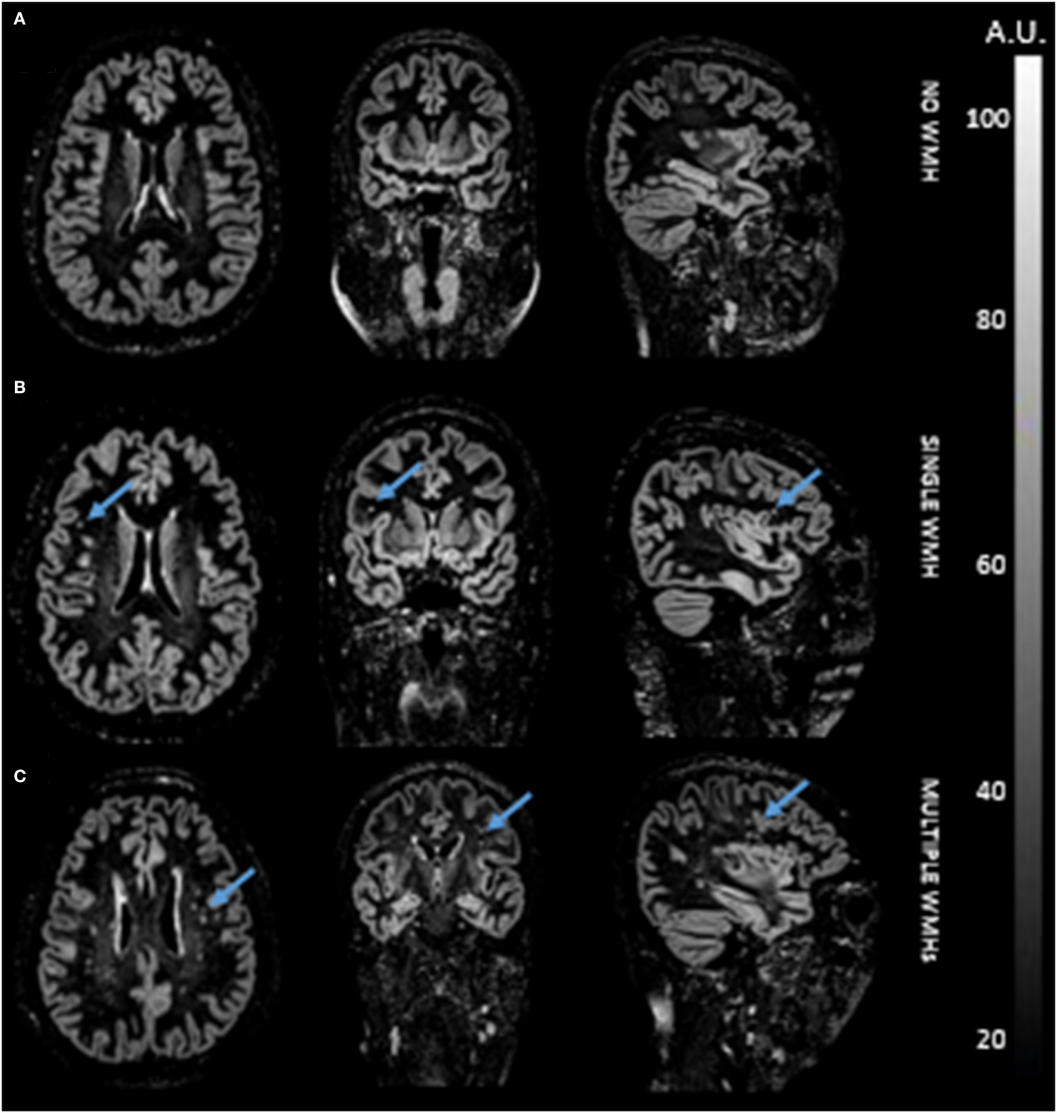
Double inversion recovery images of three representative participants without white matter hyperintensity (WMH) **(A)**, with single WMH **(B)**, and with multiple WMHs **(C)**.

The agreement between the two raters was excellent (Kappa = 0.924) (Figure 3). More WMHs were detected in the diabetic group (*p* = 0.0015). The number of WMHs is summarized in Table 3.

**Table 3:** Number of the white matter hyperintensities (WMHs) in the diabetes group and normal control groups.

No. of WMHs	No. of patients with type 2 diabetes	No. of normal control
	*N* = 36 (%)	*N* = 36 (%)
0 (Level 1)	7 (19)	22 (61)
1–3 (Level 2)	9 (25)	8 (22)
4–10 (Level 3)	8 (22)	1 (3)
11–20 (Level 4)	6 (17)	1 (3)
≥21 (Level 5)	6 (17)	4 (11)

*Numbers in parentheses represent percentages*.

### Correlation Analyses

**Figure 4:**
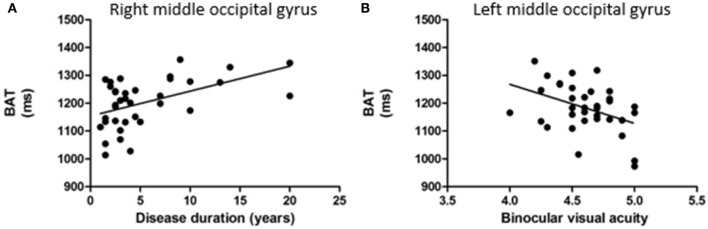
**(A)** Correlation analysis results between bolus arrival time (BAT) in the right middle occipital gyrus and the disease duration. **(B)** Correlation analysis results between BAT in the left middle occipital gyrus and the binocular visual acuity.

Within the group of type 2 diabetic patients, the BAT in the right middle occipital gyrus was significantly positively correlated with the disease duration (*r* = 0.501, *p* = 0.002) (Figure 4A); the BAT in the left middle occipital gyrus was significantly negatively correlated with the binocular visual acuity (*r* = −0.406, *p* = 0.014) (Figure 4B). No significant correlation was noted between the CBF in these brain areas, the number of WMHs and illness duration or the age at onset or MOCA scores.

## Discussion

### Hemodynamic Changes Associated with Type 2 Diabetes

In this study, we investigated the cerebral hemodynamic and WM changes of type 2 diabetes manifested in CBF, BAT, and WMH by Multi-TI ASL and DIR sequences. We found regional CBF and BAT changes in specific areas of the brain that are correlated with disease duration and binocular visual acuity. However, the mean CBF and BAT in the GM and whole brain did not show significant differences between patient and control groups, similar to previous studies (31, 32). Only the mean CBF of the deep WM showed a significant decrease in patients with type 2 diabetes.

Our results suggest that the cortical GM may be sufficiently perfused in patients with type 2 diabetes, probably due to its abundant collateral circulation. WM may be especially susceptible to declining perfusion due to its watershed location. In the previous study, hyperglycemia has been shown to lead to a wide variety of vascular abnormalities at the microvascular and macrovascular levels, including abnormal autoregulation (33), remodeling of capillaries (9), and vessel wall (34). All these changes may result in reduced perfusion and increased risk of cerebrovascular diseases.

Bolus arrival time increase in the left middle occipital gyrus, which is primarily associated with visual function (35), was detected in the diabetes group. Additionally, BAT increases were observed in the right calcarine fissure and surrounding cortex, areas also related to visual function (36). These regional BAT changes in visual areas may be linked to the observation that diabetic retinopathy is a very common complication of type 2 diabetes that has associations with the prevalence and severity of cerebral SVD (37). In a previous study of patients with diabetic retinopathy, significantly increased ADC values of the visual center (occipital gyrus) was observed (38). The correlations of prolonged BAT in visual areas with disease duration and binocular visual acuity suggest a potential mechanism of impaired visual function in diabetic patients through delayed arterial blood supply to visual areas. These BAT changes and the decrease of binocular visual acuity may be related to the secondary effect of retinopathy (37, 39). It is worth noting that although visual acuity was not assessed in control subjects in the present study, a previous study has reported normal visual acuity in a comparable group of subjects without diabetes (39) (0.03 ± 0.047 logMAR which can be converted to around 4.9–5.0 in this study).

Bolus arrival time or arterial transit time has been shown to be a valuable marker in cerebrovascular disorders such as common carotid artery occlusion (40). Increased BAT suggests the late arrival of the labeled blood, which may indicate potential increase of vascular resistance and/or redistribution of blood supply. These vascular changes may occur in patients with diabetes due to atherosclerotic plaque progression (41) and in patients with diabetic retinopathy caused by new vessel formation (42). With BAT, it is possible to measure CBF more accurately and BAT itself provides valuable cerebral hemodynamic information in patients with diabetes.

Regional CBF reduction in the left middle occipital gyrus was observed in the diabetes group compared to controls. However, after correction of PVE, the CBF findings became non-significant, suggesting that this finding may be the result of potentially concomitant atrophy of GM in diabetic patients (43). A very recent study investigated regional CBF changes of patients with type 2 diabetes and found similar CBF reductions of the visual network. They also detected widespread CBF reductions in the default mode network and cerebellum (12). However, the enrolled patients were older with longer disease duration compared to those of our study, which may explain different findings of CBF changes between the two studies. In addition, we applied BAT in the present study, which may allow the detection of early hemodynamic changes associated with type 2 diabetes.

In addition, we computed the parenchymal fraction [the ratio (GM + WM volume)/(intracranial volume)], and the mean ratio of the control group (0.7010) was slightly higher than the group of patients with diabetes (0.6903), but no significant difference was found (*p* = 0.7995). After taking this into account, the result of the group differences of global GM and local GM CBF differences did not change. The lack of significant CBF difference after PVE correction may be because the diabetes patients in our study are younger compared to the patient population of a previous study (12) and, therefore, are in a relatively early stage of diabetes.

Past studies have reported controversial findings regarding cerebral hemodynamic changes of diabetes. Some studies denied that CBF is affected by type 2 diabetes (32), nor that cognitive decline is caused by type 2 diabetes (31). Other studies reported regional CBF decrease in patients with diabetes using SPECT (10) and ASL perfusion MRI (11, 12). These different findings in literature may be due to different imaging techniques and post-processing methods employed, as well as different study populations. In previous ASL studies, only a single delay time was applied. A potential strength of our study is that we used a multi-TI ASL perfusion MRI sequence to concurrently measure BAT and CBF in patients with type 2 diabetes. Our results suggest cortical CBF may be compensated in type 2 diabetes through collateral circulation, resulting in prolonged BAT in specific brain regions.

### WM Changes Associated with Type 2 Diabetes

In the present study, 3D SPACE DIR was applied for the detection of WMHs, which has potential advantages (over standard FLAIR) for the detection of infratentorial lesions and lesions with poor contrasts on T2-weighted images (44). Because of the high GM-WM contrast, it is relatively easy to identify the location of lesions (45) with a thin slice thickness of 1.4 mm. The SPACE DIR result shows more WMHs in the diabetes group than the control group. Our findings are consistent with previous studies reporting that significantly more WM lesions were found in patients with type 2 diabetes when compared to control subjects (46).

Recent studies indicated that chronic diabetes can affect the BBB (16), thus affecting regional metabolism and microcirculation (47), leading to permanent cell damage and WM lesions (48). It may be possible that diabetes is associated with a progressive metabolic disturbance in the cerebrovascular bed that may accelerate the WM degeneration. The observed increased number of WMHs and reduced perfusion in WM in patients with type 2 diabetes is consistent with such hypothesis (49).

Past studies reported that CBF decreases in the areas of WMHs (50, 51). One caveat of the present study is that the reported reduced CBF in WM may be due to a larger number of WMHs in the patient group. We attempted to measure and compare the values of CBF within WMHs and in the residual normal appearing white matter. However, we found that the size of most of the WMHs is too small (with diameter ≤5 mm), thus does not allow accurate measurements of CBF within small ROIs. In addition, the WM mask we used mostly covers deep WM regions while WMHs are spread across WM with few overlaps with the WM mask. Nevertheless, we measured WM CBF by excluding manually drawn ROIs of WMHs within the WM mask, and the reported results of WM CBF reduction in diabetes patients compared to controls remained significant (*p* < 0.0001).

### Limitations of the Study

We note three limitations of our study. First of all, the number of enrolled subjects is relatively small compared to published studies (10, 12, 31, 32). Second, the cognitive assessment (MOCA) is relatively simple and more comprehensive evaluations need to be performed in future studies. Third, we cannot completely rule out effects of concomitant diseases such as hypertension and hyperlipidemia, although there is no significant difference in their presence between the diabetic and control groups. The inclusion of hypertension and hyperlipidemia as covariates in our analyses did not change the reported findings. Nevertheless, a strong interaction of diabetes and hypertension has been reported by a previous study (52). In future studies with large sample size, it is possible to adjust for the effect of hypertension and/or hyperlipidemia (53).

## Conclusion

In conclusion, using a multi-TI ASL technique, we observed significant differences in WM CBF and regional BAT as well as WMHs in patients with type 2 diabetes compared to normal controls. These hemodynamic differences and WM changes may be related to the complications (especially impaired vision) and risk for developing cerebral SVDs in patients with type 2 diabetes.

## Ethics Statement

The study was approved by the Medical ethics committee of affiliated Provincial Hospital of Shandong University. All patients provided written informed consent.

## Author Contributions

YS, DJJW, BZ, LY, and KJ designed the study; YS, JW, BW, LY, and KJ performed experiments; YS, JW, BW, LY, and KJ collected and analyzed data; YS, DJJW, LY, and KJ wrote the manuscript; DJJW, BZ, PJ, TQ, LY, GW, and KJ gave technical support and conceptual advice.

## Conflict of Interest Statement

The authors declare that there is no conflict of interest associated with this manuscript. Publication is approved by all authors and explicitly by the responsible authorities where the work was carried out.

## Footnotes

^1^http://www.fil.ion.ucl.ac.uk/spm/.

^2^http://www.alivelearn.net/xjview/.
